# Optimization of stool culture protocol in a tertiary hospital in Bangkok, Thailand: a prospective cohort study

**DOI:** 10.1007/s10096-026-05554-w

**Published:** 2026-05-26

**Authors:** Nanapol Ongworawut, Usa Kijsinthopchai, Samaporn Yongyod, Thidarat Wensanthia, Pawinee Kumpiranon, Pensiri Disthaporn, Yizhe Song, Korakrit Imwattana

**Affiliations:** 1 Department of Microbiology, Faculty of Medicine Siriraj Hospital, Mahidol University, Bangkok, Thailand; 2 Department of Medicine, Washington University in St. Louis, St. Louis, MO USA; 3 Department of Pathology, Texas Children’s Hospital, Houston, TX USA; 4 Department of Pathology & Immunology, Baylor College of Medicine, Houston, TX USA

**Keywords:** Bacterial diarrhea, Culture, Thailand, Bayesian latent class analysis, Limit of detection

## Abstract

**Purpose:**

Stool culture protocols are resource-intensive, and the necessity of specific selective media is frequently debated. We aimed to determine the prevalence of bacterial enteric pathogens in a Thai tertiary hospital and optimize routine culture protocols by evaluating various diagnostic methods.

**Methods:**

We evaluated 597 diarrheal stool samples using standard protocols for *Salmonella enterica*, *Shigella* spp., *Vibrio* spp., *Aeromonas* spp., and *Plesiomonas shigelloides*. Additional protocols evaluated the detection of *Campylobacter* spp. (selective medium and membrane filtration) and enteric *Yersinia* spp. (cefsulodin-irgasan-novobiocin [CIN] agar). To estimate *S. enterica* method sensitivities, we applied Bayesian latent class analysis (LCA). Additionally, analytical limit of detection (LOD) studies using spiked stool matrices were performed for *S. enterica*, *Campylobacter* spp., and *Yersinia* spp.

**Results:**

Enteric pathogens were isolated from 12.7% of the samples, predominantly *S. enterica* (10.4%) and *Campylobacter* spp. (2.2%); no *Yersinia* spp. were detected. For *S. enterica*, LCA estimated sensitivities of Gram-negative (GN) broth (97.4%) and modified semisolid Rappaport Vassiliadis (MSRV) agar (89.4%), significantly outperforming other solid media. *Campylobacter* membrane filtration and selective agar showed near-perfect clinical agreement (99.8%); however, spiked LOD testing revealed that selective agar was 10^4^-fold more analytically sensitive. For *Yersinia* spp., both CIN and MAC agars achieved a LOD of 10^4^ CFU/ml in fresh stool.

**Conclusion:**

GN broth and MSRV agar are needed to maximize *S. enterica* yield. Routine CIN agar for *Yersinia* spp. is not cost-efficient, and *Campylobacter* selective agar should replace membrane filtration to prevent false-negative reporting in low-shedding patients.

## Introduction

Bacterial culture is an important tool for the diagnosis of bacterial infections. Among the various clinical specimens, culturing bacteria from stool specimens is different because stools contain a large quantity of colonic microbiota, making it impossible and inappropriate to report every bacterium detected. Instead, only a few pathogenic species are reported [1, 2]. The list differs among different laboratories, and five pathogenic bacteria are routinely reported by the Department of Microbiology, Faculty of Medicine Siriraj Hospital, Mahidol University (in alphabetical order): *Aeromonas* spp., *Plesiomonas shigelloides*, *Salmonella enterica*, *Shigella* spp., and *Vibrio* spp., using five culture media: MacConkey (MAC) agar, thiosulfate citrate bile salts sucrose (TCBS) agar, Hektoen-Enteric (HE) agar, modified semisolid Rappaport Vassiliadis (MSRV) agar, and Gram Negative (GN) broth (see Methods below). Additionally, *Campylobacter* spp. can be tested using the membrane filtration method if requested by the attending physician. As a part of the continuous quality improvement (CQI) scheme, the Department of Microbiology sought to improve the protocol for stool culture, revisiting the current list of culture media and reported organisms. In particular, the Department proposed that using MSRV agar may not be necessary, as *S. enterica* can be isolated from several other types of culture media. Additionally, it was discussed that the culture for *Campylobacter* spp. should be routinely performed rather than per request and that the method should be improved to allow for effective detection of *Yersinia* spp. (primarily *Y. enterocolitica* and *Y. pseudotuberculosis*), in keeping with the international recommendation [1].

In the past, the exclusion of *Campylobacter* spp. and *Yersinia* spp. from the routine list of pathogenic bacteria from the stool culture was based on a current belief that the prevalence of these organisms is low. However, there has been no formal prevalence study in Thailand. A recent review showed that campylobacteriosis remains very prevalent, especially in many Asian countries [3]. This warrants a proper investigation in Thailand.

There are two main methods for the culturing of *Campylobacter* spp.: the membrane filtration method and the use of selective agar (*Campylobacter* selective agar) [2, 4]. The membrane filtration method is inexpensive but time-consuming and labor-intensive, requiring manual placement and removal of the filter paper and the initial inoculation of at least 30 min [2]. It is currently the method used at the Department of Microbiology due to its low cost. The culturing of *Yersinia* spp. can be performed using either the MAC agar at 25 °C or with a selective agar, such as cefsulodin-irgasan-novobiocin (CIN) agar.

This study aims to determine the prevalence of pathogenic bacteria in patients with diarrhea, as well as to compare different culture methods for their efficacy and cost. The results will then be used to improve the laboratory protocol for stool culture to provide the most accurate results to clinicians using the most cost-effective methods.

## Materials and methods

### Study design and clinical specimens

This prospective descriptive study was conducted at the Faculty of Medicine Siriraj Hospital, Mahidol University, a 2,000-bed tertiary hospital in Bangkok, Thailand. A total of 597 stool samples collected between March and May 2024 were included in the study. All stool specimens were submitted from patients presenting with acute diarrhea, collected in sterile containers without preservatives and transported at room temperature. To ensure organism viability, all specimens were processed within 4 h of collection. Upon reception, samples were visually categorized as liquid (Bristol type 7), unformed (Bristol type 5–6), or formed, and the presence of gross blood was noted.

Routine culture and identification.

**Fig. 1:**
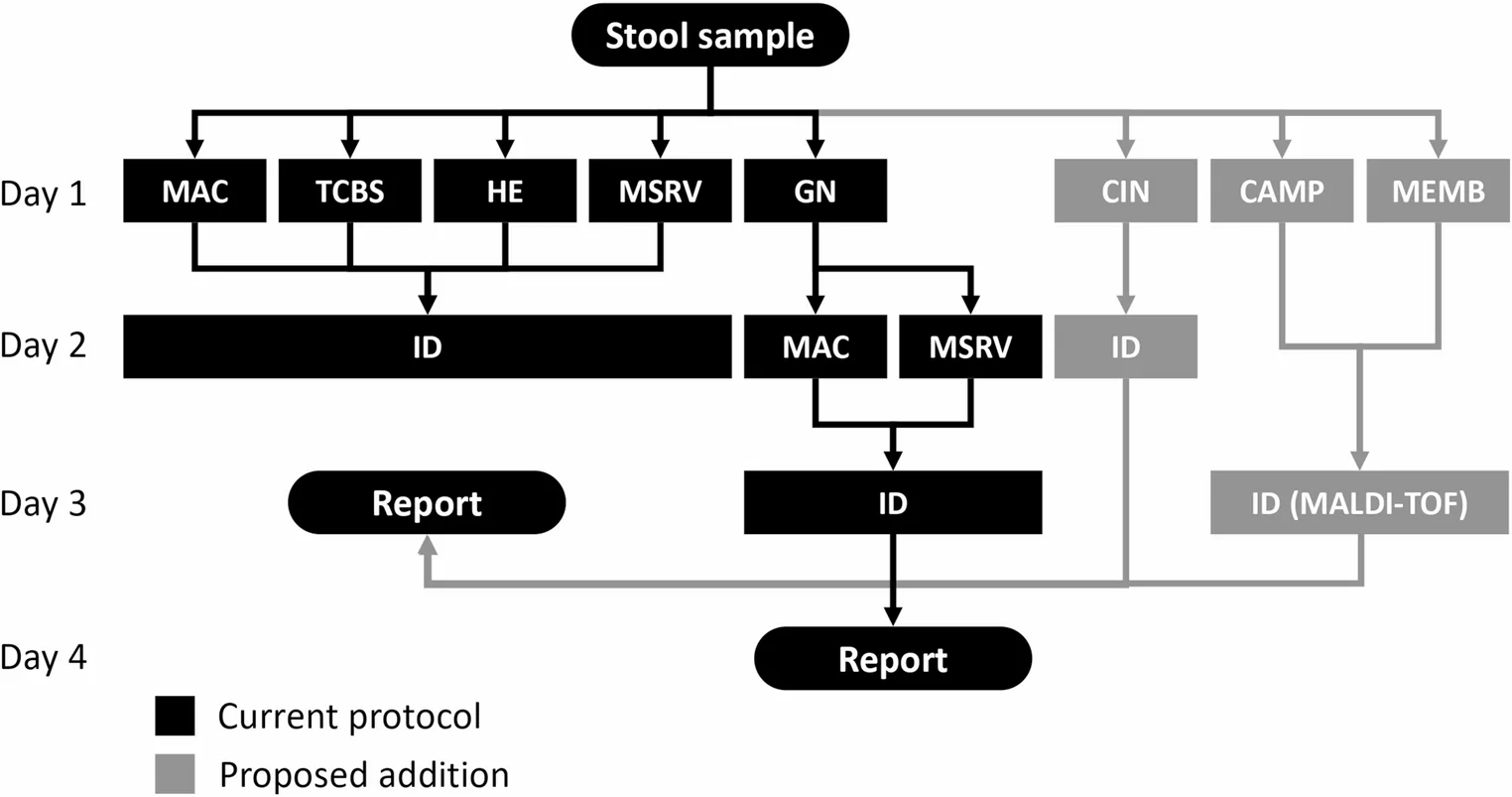
Summary of stool culture protocol. MAC; MacConkey agar, TCBS; thiosulfate-citrate-bile salt-sucrose agar, HE; Hektoen enteric agar, MSRV; modified semisolid Rappaport Vassiliadis agar, GN; Gram-negative broth, CIN; cefsulodin-irgasan-novobiocin agar, CAMP; *Campylobacter* agar, MEMB; membrane filtration method (with blood agar), ID; identification, MALDI-TOF; matrix-assisted laser desorption ionization time-of-flight mass spectrometry

All stool samples were processed according to the standard laboratory protocol. Briefly, each sample was inoculated directly onto MAC (Becton Dickinson, USA), TCBS (Becton Dickinson, USA), HE (Thermo Fisher Scientific, USA), and MSRV (Thermo Fisher Scientific, USA) plates, as well as a tube of GN broth (Becton Dickinson, USA). The first three plates were incubated aerobically at 35 ± 2 °C, in accordance with standard clinical microbiology guidelines for the recovery of enteric pathogens [1, 2], and MSRV was incubated at 42 °C. Plates were inspected for growth at 24 h. The GN broth was incubated aerobically at 35 ± 2 °C, and subcultures were performed from this single broth onto MAC and MSRV plates at 6 and 24 h of incubation, respectively. This process aims to detect five groups of enteric pathogens: *Aeromonas* spp., *P. shigelloides*, *S. enterica*, *Shigella* spp., and *Vibrio* spp. The stool culture protocol is visualized in Fig. 1. The presence of contaminating intestinal microbiota was defined by the visual growth of non-target colonies on the respective media.

### Detection of additional enteric pathogens

Besides the routine culture protocol described above, each stool sample was tested for the presence of *Campylobacter* spp. and enteropathogenic *Yersinia* spp. For *Campylobacter* spp., the stool specimen was inoculated onto a commercial *Campylobacter* selective agar (Thermo Fisher Scientific, USA), and incubated in a microaerophilic condition at 35 ± 2 °C, to also allow for the recovery of less common *Campylobacter* spp. (e.g., *C. fetus*) and *Arcobacter* spp. [5], for 48 h before inspection. The stool was also processed using a membrane filtration method with a 0.45-µm cellulose ester filter on a blood agar plate (Biomedia, Thailand) and incubated in the same manner [2]. The microaerophilic condition was achieved using CampyGen™ sachet (ThermoFisher Scientific, USA) in a 2.5 L Oxoid Anaerobic jar (ThermoFisher Scientific, USA). The final oxygen concentration was 5% according to the manufacturer’s protocol.

For enteropathogenic *Yersinia* spp., each stool sample was incubated onto the CIN (Thermo Fisher Scientific, USA) and MAC agar, and incubated aerobically at 35 ± 2 °C for 24 h before inspection.

### Species identification

Species identification was performed for suspected enteric pathogens as summarized in Table 1. For *Campylobacter* spp., colony morphology was observed on both *Campylobacter* and blood agar plates. On both plates, *Campylobacter* spp. appeared as medium-sized, wet-looking, irregular colonies. The oxidase test and Gram stain were performed on suspicious colonies. All oxidase-positive, Gram-negative curved bacilli were presumed to be *Campylobacter* spp. For the membrane filtration method, only colonies within the margin of the filtration membrane were inspected. Species confirmation was performed using the matrix-assisted laser desorption ionization time-of-flight mass spectrometry (MALDI-TOF MS, Bruker Daltonics, Germany). A spectral score of 2.00 or higher was required for species-level identification. While MALDI-TOF MS offers rapid identification, our laboratory currently utilizes it selectively for fastidious organisms like *Campylobacter* spp. to optimize cost-efficiency. Identification of other organisms (*Enterobacterales*, *Vibrio* spp., and *Aeromonas* spp.) was performed using *in house* phenotypic panels [2].

**Table 1:** Identification scheme for common enteric pathogens

Pathogens	Media	Colony characteristics	Identification
*Aeromonas*	MAC agar	Colorless colonies, oxidase positive	Biochemical tests
*Campylobacter*	Blood agar	Any colonies, oxidase positive	MALDI-TOF
	*Campylobacter* agar	Flat gray colonies, oxidase positive	MALDI-TOF
*P. shigelloides*	MAC agar	Colorless colonies, oxidase positive	Biochemical tests
*S. enterica*	HE agar	Black colonies	Biochemical tests
	MAC agar	Colorless colonies, oxidase negative	Biochemical tests
	MSRV plate	Motile colonies	Biochemical tests
*Shigella*	HE agar	Colorless colonies	Biochemical tests
	MAC agar	Colorless colonies, oxidase negative	Biochemical tests
*Vibrio*	MAC agar	Colorless colonies, oxidase positive	Biochemical tests
	TCBS agar	Any colonies	Biochemical tests
Yersinia*	MAC agar	Colorless colonies, oxidase negative	Biochemical tests
	CIN agar	Red bull’s-eye colonies	Biochemical tests

* *Y. enterocolitica* and *Y. pseudotuberculosis*; biochemical tests for *Enterobacterales* organisms include indole, triple sugar iron, lysine decarboxylase, lysine deaminase, motility, Voges-Proskauer, urease, assimilation of mannitol/malonate/citrate; biochemical tests for *Aeromonas*/*Vibrio* include arginine decarboxylase, ornithine decarboxylase, growth in 0% and 7% NaCl, cystine tryptic agar with arabinose/inositol/lactose/maltose/mannitol/salicin/sucrose

### Determination of analytical sensitivity

The analytical sensitivity was determined for the culture of *Yersinia* spp., *Campylobacter* spp., and *S. enterica*, the three organisms for which the protocols were expected to be amended, using a spiked stool method. Briefly, a 0.5 McFarland suspension of a well-characterized local isolate of *Y. enterocolitica*, *C. jejuni*, and *S. enterica* was prepared (equivalent to 10^8^ CFU/ml). Each suspension was then diluted from 10^7^ to 10^2^ CFU/ml. One milliliter of each dilution was then inoculated into 9 ml of sterilized stool (to assess the ideal sensitivity) and fresh stool (to assess a real-world sensitivity) derived from a pool of negative stool specimens, resulting in the final concentration of 10^6^ to 10^1^ CFU/ml of stool. Each stool was cultured as described above in three biological replicates. Analytical sensitivity was defined as the lowest concentration at which the culture was positive in all three repeats.

### Bayesian latent class analysis

Due to the absence of a perfect gold standard, a Bayesian latent class analysis (LCA) was performed to estimate the diagnostic sensitivity and specificity for the *Salmonella* culture methods [6]. The model assumed two latent classes (‘infected’ vs. ‘not infected’) based on the pattern of results from four indicators: MAC, HE, MSRV, and GN broth enrichment (defined as positive if subsequent subculture to MAC or MSRV yielded growth). The analysis was conducted using the randomLCA package in R version 4.5.2 (R Core Team, Vienna, Austria), utilizing a probit link function. Conditional independence was assumed between the distinct culture media types.

### Statistical analysis

Apart from the LCA, all other statistical analyses were performed using Python version 3.13.9 (Python Software Foundation, Wilmington, DE, USA). Categorical data were reported using proportions with a 95% confidence interval (95% CI) calculated using the Wilson method. Comparison between different methods was performed using either chi-squared or Fisher’s exact test as appropriate. Statistical significance was achieved at *p* < 0.05.

## Results

### Prevalence of enteric pathogens

A total of 597 stool samples were included in this study; 176 (29.5%) were liquid (the sample immediately takes the shape of the container, equivalent to Bristol stool type 7), while 421 (70.5%) were unformed (the sample maintains its shape but gradually takes the shape of the container, equivalent to Bristol stool type 5 or 6). Seventeen samples (2.8%) were visibly bloody. Enteric pathogens were isolated in 76 (12.7%) samples, more frequently from unformed stool samples (64 of 421; 15.2%) than liquid stool samples (12 of 176; 6.8%) (*p* = 0.005, Cramér’s V = 0.115), but no significant difference between bloody and non-bloody stool samples (1/17 [5.9%] vs. 75/580 [12.9%], *p* = 0.710). The prevalence of each enteric pathogen is summarized in Table 2. The organism with the highest prevalence was *S. enterica* (10.4%), followed by *Campylobacter* spp. (2.2%). Despite using two agar plates (MAC and CIN), no *Yersinia* spp. was detected in this population.

**Table 2:** Prevalence of enteric pathogens

Organism	Number (%)	95% CI
*Aeromonas* spp.	8 (1.3%)	0.5–2.6%
*Campylobacter* spp.	13 (2.2%)	1.2–3.7%
*P. shigelloides*	8 (1.3%)	0.5–2.6%
*Salmonella enterica*	62 (10.4%)	8.1–13.1%
*Shigella* spp.	0 (0.0%)	0.0–0.6%
*Vibrio* spp.	3 (0.5%)	0.1–1.5%
*Yersinia* spp.	0 (0.0%)	0.0–0.6%
Stools with more than 1 pathogens	8 (1.3%)	0.5–2.6%
Total positive	76 (12.7%)	10.1–15.7%

### Comparison of detection methods for *Campylobacter* spp.

Two culture methods for *Campylobacter* spp. were evaluated: *Campylobacter* selective agar and membrane filtration. The total number of positive samples was 13 by *Campylobacter* selective agar and 12 by membrane filtration. Only *C. jejuni* (10, 76.9%) and *C. coli* (3, 23.1%) were detected in this population. The percent agreement was 99.8%, with only one disagreement, and Cohen’s kappa was 0.959 (near perfect agreement). However, the presence of intestinal microbiota was significantly higher with the *Campylobacter* agar method (538/597 [90.1%] vs. 4/597 [0.7%], *p* < 0.001).

### Comparison of detection methods for *S. enterica*

The detection of *S. enterica* was performed using 3 plates (MAC, HE, and MSRV) and a GN broth enrichment method (further subcultured onto MAC and MSRV), and the performance of each culture medium was compared. A total of 62 stool samples were positive for *S. enterica*, 49 (79.0%) were positive on at least 1 of the 3 plates, while 13 (21.0%) were only positive in the GN broth. Among the 49 stool samples positive on the plates, 21 (42.8%) were positive only on the MSRV plate, 3 (6.1%) of which would also later be negative in the GN broth (Table 3).

**Table 3:** Comparison of detection methods for *S. enterica*

Methods	Cost (US$)	All positive	Exclusively positive*	Sensitivity	Specificity
MSRV	0.07	45 (72.6%)	2 (3.2%)	89.4%	99.5%
HE	0.50	27 (43.5%)	0 (0.0%)	58.0%	100.0%
MAC	0.18	22 (35.5%)	0 (0.0%)	47.7%	100.0%
GN + MSRV	0.08	57 (91.9%)	13 (21.0%)	97.4%	97.8%
GN + MAC	0.19	18 (29.0%)	0 (0.0%)		

percentage of total (*n* = 62), * exclusively positive refers to the number of specimens that *S. enterica* was isolated only with this method. Sensitivity and specificity were derived from the Bayesian latent class analysis

### Diagnostic sensitivity and specificity of the *S. enterica* culture methods

Based on the Bayesian latent class analysis, the prevalence of salmonellosis in the study population was 8.1%, at the lower margin of the 95% confidence interval of the actual prevalence of *S. enterica* positivity (Table 2). The GN broth enrichment method had the highest sensitivity (97.4%), followed closely by direct MSRV inoculation (89.4%), while HE and MAC had low sensitivity (58.0% and 47.7%, respectively). The specificity was similarly high for all methods (up to 100.0%, Table 3).

### Analytical sensitivity of the culture methods

**Fig. 2:**
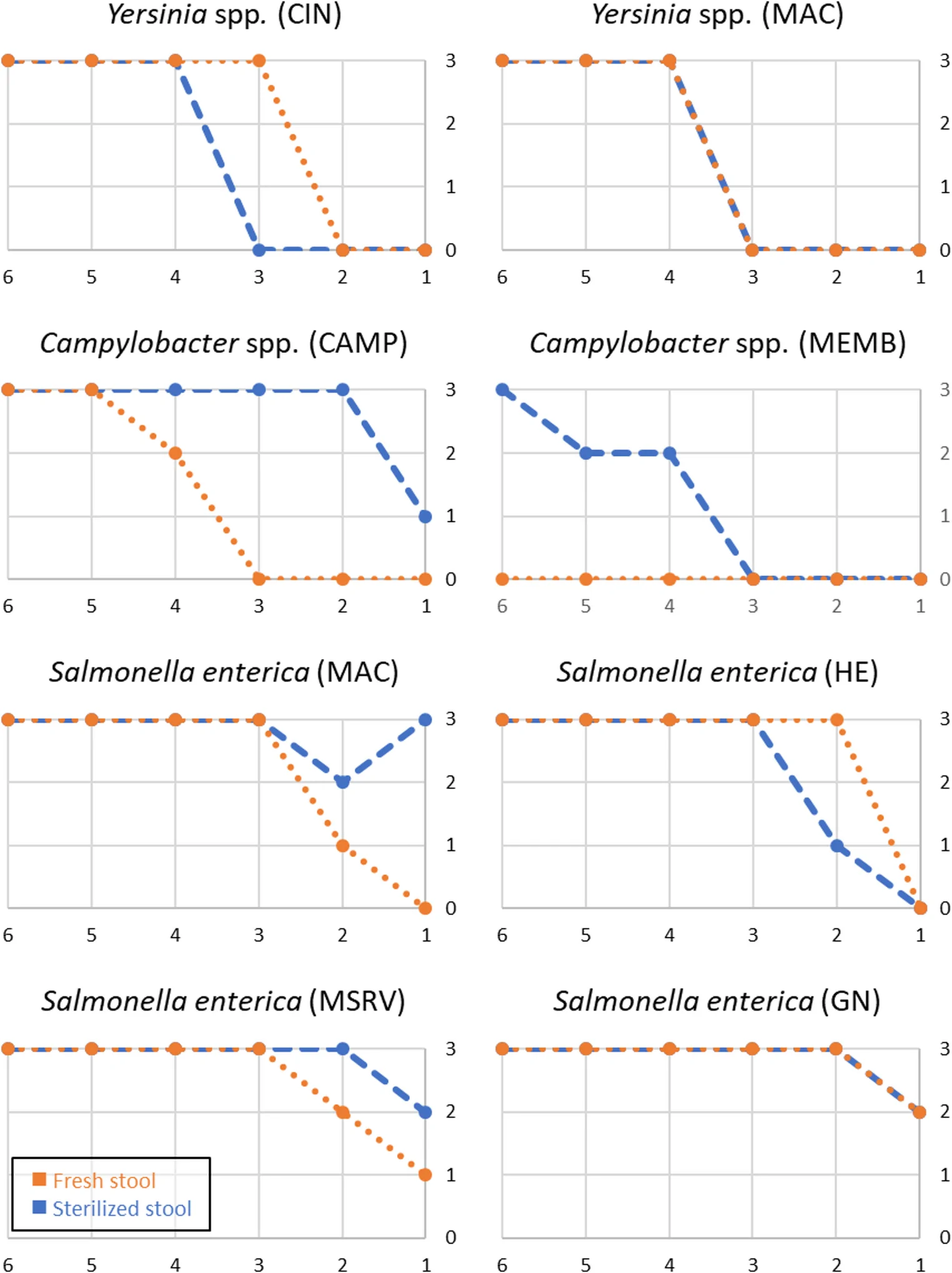
Analytical sensitivity of each culture method. The X-axis denotes the expected organism concentration in log_10_ CFU/ml. The Y-axis denotes the number of repeats that yielded culture confirmation. CIN; cefsulodin-irgasan-novobiocin agar, MAC; MacConkey agar, CAMP; Campylobacter agar, MEMB; membrane filtration method (with blood agar), HE; Hektoen enteric agar, MSRV; modified semisolid Rappaport Vassiliadis agar, GN; Gram-negative broth

All agar-based culture methods for *Yersinia* spp. and *S. enterica* could reliably detect organisms as low as 10^3^ CFU/ml of stool, with only up to 1 dilution difference between fresh and sterilized stool. GN broth enrichment culture could detect *S. enterica* as low as 10^2^ CFU/ml, at least one dilution lower than the agar-based culture. *Campylobacter* selective agar was at least 10^4^ times more sensitive than the membrane filtration method, with sterilized stool having at least 10^3^ times higher sensitivity than fresh stool (Fig. 2).

## Discussion

Stool culture is perhaps the most complicated bacterial culture process [1], as stool samples contain numerous commensal species, many of which are similar to true enteric pathogens. This warrants the use of several selective and differential culture media to help isolate the pathogens. Despite the effort, a few pathogens, such as *Campylobacter* spp. and *Yersinia* spp., were not routinely tested for, as they require specific culture conditions not routinely performed, and the anecdotal belief that they are not prevalent [1, 7]. On the other hand, the notoriety of *S. enterica* and its ability to cause disease at a relatively low infectious dose resulted in the inclusion of 4 different culture media in the culture protocol to ensure that all cases of *S. enterica* infection are detected [1], although the necessity of each medium has never been assessed. This study aims to evaluate the current stool culture protocol and make necessary adjustments to improve the efficiency of the method.

The overall prevalence of enteric pathogens in this study is substantially lower than a historical study in 1983 [8]. Although these two studies are not representative of the whole country, the reduction of prevalence likely indicates the improvement of sanitation and public health systems in the country during the past 40 years. The high prevalence of *S. enterica* (10.4%) and *Campylobacter* spp. (2.2%), in keeping with a recent study [9], could partially be explained by the improvement of stool culture methods [1]. Also, these two organisms have zoonotic potential [3, 10]. Thus, the rise in the prevalence of these organisms can also be accounted for by the concurrent rise of zoonotic diseases [11].

Notably, the prevalence of positive stool culture was more than twice as high in unformed stool compared to liquid stool samples (15.2% vs. 6.8%, *p* = 0.008). Liquid stool may be more associated with food intoxication and viral gastroenteritis, both of which would likely provide negative stool culture results [12, 13]. However, this study did not perform additional tests (either molecular tests or antigen detection) to confirm the diagnosis of viral gastroenteritis, nor was the data on the final diagnoses included in the study.

*Campylobacter* spp. was the second most prevalent enteric pathogen in this study, the prevalence of which was also higher than in the previous study [1], refuting the current belief and in keeping with the prevalence in other Asian countries [3]. Thus, the stool culture protocol must be amended to also include the detection of *Campylobacter* spp. Two methods were evaluated in this study. The conventional membrane filtration method is relatively cheaper (US$1.51 vs. US$2.82, based on the 2024 currency conversion rate), although it involves additional steps and is more labor-intensive. The *Campylobacter* selective agar method is simple, but the agar itself is more expensive [4]. There was an almost perfect correlation in the detection of *Campylobacter* spp. between the 2 methods, similar to a previous study [14]. However, the membrane filtration method was superior in removing the contaminating commensal bacteria (contamination 0.7% vs. 90.1%, *p* < 0.001). On the other hand, the analytical sensitivity of the *Campylobacter* selective agar was deemed to be at least 10^4^ times higher than that of the membrane filtration method (Fig. 2). In the presence of commensal bacteria (fresh stool protocol), the limit of detection of the membrane filtration method was higher than 10^6^. This difference in the analytical sensitivity contrasts with the near-perfect correlation of the two methods in this study cohort. This suggests that patients with campylobacteriosis generally shed massive amounts of bacteria in the stool, despite the low infectious dose [15]. The use of the membrane filtration method could also detect less common *Campylobacter* spp. inhibited by the selective media, such as *C. upsaliensis* [5]. Although the membrane filtration method is less expensive and successfully detected acute, potentially higher-burden infections in our cohort, its severe analytical penalty (10^4^-fold reduction in sensitivity) poses an unacceptable risk of false-negativity in patients with lower bacterial burdens. Therefore, we conclude that direct plating on selective agar is the clinically superior and necessary method for routine diagnostics.

Despite the effort, no *Yersinia* spp. was detected in this study. The organism was also the least prevalent in the previous study [8], which was also lower than the estimated global prevalence [16]. The analytical sensitivity analysis determined that both CIN and MAC agars could detect as low as 10^4^ CFU/ml of organisms in the stool, comparable to the sensitivity of *S. enterica* detection. This suggests that it is not a common pathogen in the country. Thus, the inclusion of CIN agar (US$ 0.86 per plate) for the specific detection of *Yersinia* spp. may not be cost-efficient in this population. Alternatively, *Yersinia* spp. could be detected on MAC agar, which is already included in the protocol, and the detection could be enhanced by keeping the MAC agar plate at room temperature for another 24 h [2], further diminishing the need for CIN agar in the stool culture protocol. This would also have very little effect on the overall workflow, as *Campylobacter* spp. requires at least a 48-hour incubation time. Thus, the prolonged incubation of MAC at room temperature would not delay the turnaround time (Fig. 1).

In the current stool culture protocol, 3 agar plates and a GN broth were used for the detection of *S. enterica*, and the organism was the most prevalent pathogen in the study, with a higher prevalence than all other organisms combined. This study aims to determine whether any culture media, especially MSRV plate and GN broth, are redundant. The removal of the MSRV plate from the protocol would delay the detection of *S. enterica* in 18 (29.0%) samples by at least 24 h and would miss the detection in 3 (4.8%) samples. The delay is due to the nature of broth culture, as a single colony cannot be isolated from the broth, requiring a subculture on an agar plate and additional 1-day incubation. Removal of the GN broth would miss the detection of *S. enterica* in 13 (21.0%) samples. Indeed, the missed samples were likely to have fewer *S. enterica*, as determined by the analytical sensitivity analysis. However, further study is needed to determine whether this microbiological difference is also translated into differences in clinical outcomes. Based on the current results, all culture media should be retained in the stool culture protocol to ensure the detection of *S. enterica*.

This study has several limitations. First, it was conducted at a single tertiary hospital in a metropolitan area; thus, the prevalence reported in this study may not perfectly reflect community or rural settings. Second, a three-month sampling period precludes the analysis of seasonal variations. However, unlike temperate zones, where enteric pathogens often exhibit seasonal variability, enteric pathogens are highly endemic year-round in tropical regions like Thailand [17]. Therefore, this timeframe provides a robust representative cross-section of the baseline infection burden. Third, this study did not explicitly evaluate Shiga toxin-producing *Escherichia coli* (STEC). Nevertheless, our validation of GN broth serves a dual purpose: in addition to maximizing *S. enterica* recovery, GN broth is also internationally recommended as an ideal enrichment matrix for the direct detection of Shiga toxins via enzyme immunoassay or polymerase chain reaction [1, 18]. Consequently, our optimized protocol can be seamlessly adapted to include STEC screening without introducing additional primary culture media. Finally, our strict reliance on bacterial culture limits our ability to report on the complete epidemiology of diarrhea, including viral and parasitic etiologies.

In conclusion, there was a marked increase in the prevalence of bacterial enteric pathogens in Thailand, with *S. enterica* and *Campylobacter* spp. as the most common pathogens. The high prevalence of *Campylobacter* spp. warrants the inclusion of a specific method of detection in the stool culture protocol. Given the rarity of *Yersinia* spp. in this cohort, inclusion of a specific selective medium may not be necessary in this population at present, though surveillance should continue in case of epidemiological shifts. Several culture media, including the MSRV plate and the GN broth, are needed for the highest sensitivity of the detection of *S. enterica*.

## Data Availability

No datasets were generated or analysed during the current study.
